# Epidemiological and Operational Constraints on the Feasibility of Peste des Petits Ruminants Eradication Target by 2030: Necessity for a New Pragmatic Road Map

**DOI:** 10.1155/tbed/4504556

**Published:** 2026-08-24

**Authors:** Kadir Yeşilbağ, Mevlüt Yaşar

**Affiliations:** ^1^ Department of Virology, Faculty of Veterinary Medicine, Bursa Uludag University, Bursa, Türkiye, uludag.edu.tr

**Keywords:** constraints, eradication, feasibility, PPR, surveillance

## Abstract

Peste des petits ruminants (PPR) remains a major constraint to small ruminant production and food security, and a global eradication target for 2030 have been set by FAO/WOAH. This review analyses recent evidence on the feasibility of that target by integrating epidemiological indicators, operational performance of vaccination and surveillance systems, and structural drivers that sustain transmission. We find that the main barriers to eradication are not either virological/methodological or programmatic: achieving and sustaining high population immunity in pastoral and highly mobile systems; cold‐chain and logistics constraints that reduce effective vaccine delivery; uneven surveillance sensitivity, timeliness, and data quality that limit detection of residual foci; and persistent reseeding via informal trade and transboundary movements. Multihost dynamics at wildlife–livestock interfaces, together with fragility factors such as conflict and climate‐driven mobility, further complicate the endgame. Recent incursions into previously free territories highlight that progress is reversible when borders and surveillance systems are stressed. The dominance of PPR virus (PPRV) lineage IV in global spatiotemporal distribution has become increasingly evident in the last 10 years. We conclude that global freedom by 2030 is unlikely without rapid, synchronized improvements in coverage, surveillance performance, and cross‐border coordination. A pragmatic pathway beyond 2030 should prioritize risk‐based vaccination targeting high‐connectivity corridors and markets, routine postvaccination seromonitoring as a decision trigger, strengthened endgame‐grade surveillance (including targeted genomic investigation), and regional compacts that align implementation with broader resilience and economic development goals. We suggest that a new, revised global planning consisting of three consecutive phases would be more appropriate and predictable.

## 1. Introduction

Peste des petits ruminants virus (PPRV), a single‐stranded, negative‐polarity RNA virus, currently classified as *Morbillivirus caprinae*, is a member of the genus *Morbillivirus* within the family Paramyxoviridae [1]. PPR, due to its high morbidity and mortality rate in sheep and goats, leads to significant losses in small ruminant production and negatively impacts income stability and food security, particularly in low‐income producer communities [2, 3]. The acute clinical presentation of the disease, combined with its potential for rapid herd‐level spread, increases control costs, and recurring outbreaks can create persistent economic pressure in endemic areas, leading to losses in household income and significant decreases in farm profitability [4]. Beyond its devastating clinical impacts, PPR is now recognized as a significant threat to global food security, with updated estimates indicating annual economic losses of approximately USD 2.1 billion and affecting the livelihoods of over 330 million smallholder farmers [5]. Consequently, WOAH and FAO have recently emphasized that PPR eradication should be recognized as an international public good, requiring political commitment and resource mobilization beyond national borders to support a coordinated global effort. [5]. Although only one serotype of PPRV has been identified antigenically, phylogenetic analyses based on the nucleoprotein (N) and fusion (F) genes have distinguished four genetic lineages (I–IV) [6, 7]. Lineages I–III have historically been reported in Africa, while lineage IV was long associated predominantly with the Middle East and Asia; however, in the last 20 years, lineage IV has been increasingly detected in Africa and is beginning to replace other lineages in some regions [6, 8, 9]. Following the global declaration of freedom from rinderpest in 2011, FAO and WOAH launched the Global Control and Eradication Strategy for PPR (PPR GCES) in 2015, adopting the goal of global eradication of PPR by 2030 [3, 10]. The strategy, developed based on the experience of rinderpest eradication, presents the following as key technical justifications in favor of PPR eradication: the limited natural host range of PPR, its transmission through direct contact and respiratory secretions without vector mediation, its existence as a single serotype, the availability of live attenuated vaccines that provide strong and long‐lasting immunity, and the accessibility of current diagnostic tools [6, 11].

However, the PPR GCES developed by FAO and WOAH and extensive reviews in recent years clearly demonstrate that significant obstacles remain to progress towards the 2030 target [11, 12]. These obstacles include epidemiological issues such as transboundary animal movements and surveillance gaps, operational difficulties related to vaccination and monitoring processes, sociopolitical factors associated with political and socioeconomic instability, and the need for sustainable funding [10–12]. The Global Eradication Programme II and III (also referred to as the Blueprint), operationalized by FAO and WOAH, emphasizes that eradication must rely on a multidimensional approach integrating wildlife surveillance, gender inclusion, and youth engagement [13]. However, epidemiological developments during 2024–2025, particularly the re‐emergence and spread of PPR in parts of Europe, have renewed concerns about whether the current Blueprint is sufficient to sustain effective surveillance, movement control, and field‐level outbreak containment on the path to the 2030 target [12, 14].

This review assesses the feasibility of achieving global PPR eradication by 2030 in the light of current epidemiological, operational, and sociopolitical constraints and proposes alternative pathways beyond 2030.

## 2. Global Epidemiological Situation for PPR

### 2.1. Endemicity and Geographical Distribution

PPR is endemic to much of Africa, the Near and Middle East, and large parts of Asia, including South Asia; it has been confirmed in more than 70 countries and is reported to threaten more than 1.7 billion of the global sheep and goat population [3, 13, 15, 16]. A regional review covering the Middle East from 2017 to 2021 shows that the presence of PPR is confirmed or strongly probable in most of the 15 countries assessed [17]. Furthermore, it highlights that the true epidemiological situation is significantly underrepresented due to marked differences in surveillance capacity and reporting levels among countries [17]. It has been reported that in many countries, vaccination campaigns lack continuity, vaccination coverage falls below the level necessary for eradication, and key risk areas are not adequately targeted, preventing a significant reduction in disease incidence [3, 16, 17]. In response to this persistent endemicity, the Pan‐African Programme for the Eradication of PPR was officially launched in February 2025 in Addis Ababa, under the theme Uniting Africa for a PPR‐Free Future. Coordinated by the African Union through its technical unit, the African Union–Interafrican Bureau for Animal Resources (AU‐IBAR), the initiative has been framed around a total budget of EUR 528 million for the Pan‐African Programme for the Eradication of PPR. The European Union currently supports the first phase with EUR 8 million, as part of a planned broader contribution, and the programme aims to support economic transformation in alignment with the AU Agenda 2063 objectives in a continent where livestock contributes 30%–50% of agricultural GDP [14, 18].

### 2.2. Field Indicators From Selected Endemic Settings

According to FAO and WOAH’s assessment of the Central and East Asia and Western Eurasia region, by the end of the 2017–2021 period, only the Russian Federation had officially achieved PPR‐free status in the region, Armenia, Azerbaijan, Kazakhstan, Kyrgyzstan, Turkmenistan, and Uzbekistan had not reported any PPR cases, and Georgia and Tajikistan had not reported any outbreaks in the last 5 years [19]. These data indicate that while there is potential for progress in some countries in the region, the simultaneous progress necessary for eradication on a global scale has not yet occurred [12, 19].

A recent review focusing on Ethiopia reports a national eradication target of 2027 and highlights persistent implementation constraints, including limited veterinary access in pastoral settings, cold chain limitations, and logistical barriers [20]. Field data from Borena further indicate that herd immunity can remain below the FAO‐WOAH‐recommended 80% threshold [21]. Seroepidemiological studies conducted in pastoral and agro‐pastoral systems in Tanzania have shown that the nationwide seroprevalence is around 10% [22]. Recent data from Uganda further emphasizes the risk inequality between production systems, with seroprevalence as low as 6.1% in mixed crop‐livestock systems, while in pastoral systems, this rate is at 44.1%, indicating that these areas are high‐risk hotspots for virus circulation [23]. Similarly, a study conducted in Nile Delta, Egypt, in 2025 identified shared water resources as a critical factor and found substantially higher odds of PPRV seropositivity in animals sharing water sources [24]. A cross‐sectional serosurvey conducted in three districts in the central part of the Oromia region, Ethiopia, between 2019 and 2021 reported PPRV seropositivity at the herd level of 71.2% and ~50% at the animal level, a finding consistent with widespread exposure and intensive circulation under endemic conditions [25]. Comparable field indicators have also been reported in South Asia. This situation is also supported by the current outbreaks reported in the Punjab state of India in 2025, which have a mortality rate of 24.86%. The fact that case fatality rates in some establishments reached 57% during these outbreaks indicates that the endemic pressure and economic losses in the region remain serious [26]. In Punjab, Pakistan, a cross‐sectional serosurvey conducted in unvaccinated sheep and goats aged ≥6 months (*n* = 722) in 2024 found an overall PPRV seroprevalence of 79.77%, with higher seropositivity in goats (90.27%) than in sheep (68.75%) and marked district‐level heterogeneity [27]. Supporting this local heterogeneity, a comprehensive meta‐analysis covering 20 years (2004–2023) in Pakistan estimated the national pooled prevalence at 51%, with the Sindh province exhibiting a significantly higher endemic burden at 61% [28]. Findings from Bangladesh and Africa indicate that PPR spread is closely linked to market‐connected trade routes and cross‐border animal inflows and that seasonal pastoral herd movements and commercial shipments can drive rapid within‐country and regional dissemination [29–31].

## 3. Silent Circulation and Multihost Epidemiology

### 3.1. Subclinical Infection and a Typical Domestic Hosts

Although PPR infection in small ruminants usually presents with pronounced clinical symptoms, research continues into the potential role of subclinical infections and interspecies transmission in virus circulation in endemic regions. The epidemiological significance of large ruminants such as cattle, buffalo, and camels is a subject of debate, particularly in light of the 2030 eradication targets. While cattle are known to be resistant to clinical disease, they have been reported to be susceptible to infection and seroconversion after contact with infected small ruminants. Experimental infection studies present conflicting data regarding the transmissibility potential of these species; a previous study reported that viral antigen and nucleic acid could be detected in cattle for up to 397 days without clinical signs, suggesting that these species could be potential reservoirs [32].

In contrast, more recent experimental studies have observed that although infected cattle and camels develop seroconversion, they do not shed the virus to a level sufficient to transmit it to naive animals. Based on these findings, it has been stated that large ruminants should be considered as “dead‐end hosts” [33, 34]. These experimental data are also supported by recent comparative pathogenesis studies and molecular field surveys. For example, a comparative study comprising cattle and goats confirmed that clinical signs and virus shedding were quite limited in cattle [35]. Accordingly, the PPRV genome was detected in clinically infected sheep herds in the Marmara region of Türkiye, while viral RNA or antibodies in cattle could not be detected in the same area [36].

On the other hand, seroepidemiological studies conducted in Africa and Asia show varying seroprevalence rates in large ruminants. Data from Tanzania, Ethiopia, and India confirm that cattle and buffaloes are exposed to the virus, particularly in mixed herd systems [37–40]. The 42.36% seroprevalence rate detected in buffaloes in Bangladesh suggests that species‐specific susceptibility differences should be investigated [39]. Considering the current literature, even though large ruminants do not play a major role in the infection chain, their ability to develop seroconversion suggests that they can be used as “indicators” (sentinels) to monitor virus circulation in areas where clinical disease might be overlooked [38, 40]. Despite all this data, it is not satisfactory to fully assess the role of members of Bovidae and Camelidae in the epidemiology of PPR infection in nature.

### 3.2. Wildlife Interface and Implications for Eradication

In recent decades, expanding evidence of PPRV infection in multiple wildlife artiodactyls has highlighted the importance of the wildlife‐livestock interface for both virus persistence and feasibility of eradication [41, 42]. Serological surveys from the Northern Albertine Rift and Greater Serengeti ecosystems show widespread but generally low‐to‐moderate seroprevalence in African buffalo and various antelope species, with concurrent endemic circulation in nearby sheep and goats, indicating frequent spillover from livestock and only limited onward transmission among wild hosts under these conditions [43, 44]. Similarly, systematic reviews and meta‐analyses compiled from Asia and Africa reveal atypical hosts and a high prevalence of PPRV exposure in wildlife, pointing to a multihost circulation system and a dramatic expansion of the host range over the last 20 years [42]. Current data suggest that most free‐living large wild bovids and many antelope species are closer to being “victims” or dead‐end hosts than reservoirs and that sustainable wildlife cycles are theoretically only possible in very large and multispecies populations (e.g., Eastern Mongolia, Boma‐Gambela, and Great Serengeti) [19, 44]. However, reported severe PPR outbreaks in saiga and other wild ungulates in Mongolia have demonstrated that in certain ecosystems, wild species can act as bridge hosts for both population collapse and “spillback” to domesticated herds [15, 45].

This multihost structure demonstrates that eradication strategies based solely on sheep and goat vaccination may be insufficient, and that surveillance, diagnosis, and risk‐mapping programs that exclude wildlife risks overlook “silent” foci [15, 41, 44, 46]. Within the framework of the FAO/WOAH global strategy, validated diagnostic tests for wild species, ecosystem‐specific research, and the systematic integration of wildlife, at least as indicator (sentinel) and bridge hosts, into eradication programs are critically important [41, 46, 47]. A successful implementation of this approach has been demonstrated in Kazakhstan; the maintenance of the country’s PPR‐free status is supported by systematically negative results from more than 86,000 serological tests conducted in buffer zones [48]. In contrast, the necessity of integrating wildlife surveillance is emphasized by the fact that PPRV threatens not only livestock but also biodiversity conservation objectives [41]. This multihost transmission dynamic has recently been confirmed in the United Arab Emirates; molecular research has determined that lineage IV PPRV not only circulates in domestic herds but also causes fatal outbreaks in wild Dama Gazelles, highlighting the complexity of control at the wildlife‐domestic animal interface [49].

## 4. Programmatic Bottlenecks to Eradication

### 4.1. Vaccination Constraints: Cold Chain, Coverage, and Herd Immunity Thresholds

The backbone of the PPR eradication program consists of attenuated live PPR vaccines prepared with the strains Nigeria 75/1 (lineage II) and Sungri/96 (lineage IV); these preparations are highly immunogenic products that meet the minimum standards of WOAH (10^−2.50^–10^−3.00^ TCID_50_/dose) and provide long‐lasting, often lifelong, immunity in sheep and goats with a single dose [50, 51]. Controlled experimental studies have shown that both the Nigeria 75/1 and Sungri/96 vaccines provide sterilizing immunity against the experimental challenge with intranasal virulent PPRV after subcutaneous or intranasal administration and completely prevent clinical disease [50, 52]. Nevertheless, all of these vaccines are live and thermolabile; they require a strictly uninterrupted cold chain (2–8°C) from the production facility to the application point in the field, which creates a significant operational bottleneck in hot‐climate regions with poor infrastructure, where PPR is most prevalent [12, 16, 51]. Significant progress has been made in the field of thermostability in recent years. A comparative evaluation of 37 batches of Nigeria 75/1‐based vaccine series produced using different stabilizers and lyophilization conditions revealed that formulations containing skim milk, lactalbumin‐sucrose, and trehalose, in particular, were able to maintain the minimum titer threshold of WOAH for 3–5 days at 40°C but experienced a rapid titer loss of approximately ~1 log_10_ in the first 3 days of this process [51]. These findings indicate that batches meeting the criteria of ≥10^2.50^–10^3.00^ TCID_50_/dose titer and <1 log_10_ loss at 40°C for 5 days can be used in the field as a thermostable PPR vaccine for at least 2–3 weeks without a cold chain [51]. However, such thermostable formulations have not yet been widely integrated into operational programs; many countries still use classic, cold‐chain‐dependent lyophilized vaccines [12, 16, 51]. Similarly, next‐generation marker vaccines offering DIVA (differentiating infected from vaccinated animals) capability are also showing promise at the experimental level. Two live attenuated DIVA vaccines (Sungri/96‐DIVA and Nigeria 75/1‐DIVA), based on the replacement of the C‐terminal variable region of the PPRV N protein with dolphin morbillivirus sequences, were found safe in pilot goat studies and provided complete clinical protection comparable to the original vaccine strains against an experimental infection (challenge) model with an intranasal virulent strain [53]. The development of two novel ELISAs compatible with these vaccines, capable of differentiating between natural infection and vaccine immunity, theoretically offers the possibility of DIVA‐based surveillance and seromonitoring in the later stages of eradication [53]. However, it appears that none of these candidates have yet transitioned to large‐scale field deployment, commercial licensing, and national stock/banking mechanisms; critical steps such as production capacity, cost, and integration with field diagnostic kits have also not been completed [12, 16, 53].

Therefore, key components of the theoretically available toolset for PPR eradication in official documents and global strategies, particularly widely accessible DIVA vaccines and commonly used thermostable preparations, have not yet been scaled to the field by the 2030 perspective [12, 16, 51]. This situation maintains the risk of efficacy loss due to cold chain breaks in hot climates and keeps the logistical costs of eradication programs high.

The baseline reproduction number (*R*_0_) for PPRV varies depending on the region and production system, but most studies based on modeling and field data place *R*_0_ in the 3–6 range; this corresponds to a theoretical herd immunity threshold (1–1/*R*_0_) of ~67%–83% [54, 55]. Thus, the FAO/WOAH GCES recommends maintaining a postvaccination immunity rate (PVIR) of at least 70%–80%, and in practice, ≥80%–85% in endemic regions, with even higher coverage targeted in downwind foci [12, 16, 55–57].

However, the field data indicate significant deviations from these targets. Recent seromonitoring data from North Shewa, Ethiopia, further support this pattern, showing that although individual animal‐level antibody immunity reached 65.35%, only 22.27% of flocks achieved the ≥80% immunity threshold considered necessary for effective interruption of transmission [58]. This shortfall is consistent with operational constraints reported from the same region, including budget limitations, security‐related disruptions, lack of animal identification, absence of postvaccination seromonitoring, high herd mobility, and rapid turnover of young stock [59]. In addition, maternal antibody immunity in kids and lambs was only 52.3% and declined with age, suggesting that young animals may continue to replenish the susceptible population unless follow‐up vaccination is appropriately timed [58]. A metapopulation model fitted to national serological surveillance data in Ethiopia has revealed that the pastoral production system functions as a virus reservoir as a whole; the virus continuously “spills over” from there to sedentary herds, and even in cases where the proportion of immune individuals in at least 71% of pastoral villages can be permanently maintained above the 37% threshold, it still requires annual, high‐coverage recurring campaigns [54]. In the same context, an ex‐ante modeling analysis conducted in two smallholder production systems, representative of sheep husbandry in semi‐arid areas and goat husbandry in humid zones, indicated that PVIR declines rapidly over a 4‐year program as a consequence of population turnover and demographic processes. The analysis further showed that interruption of transmission requires targeting the fully eligible population, particularly during the first 2 years, and implementing vaccination campaigns achieving at least 80% coverage [56]. Field seromonitoring studies also support this pattern. In West Gojjam, Ethiopia, postvaccination herd immunity measured 4 months after vaccination of sheep flocks with a Nigeria 75/1‐based vaccine was 76.7%, which is slightly below the 80% threshold recommended by the global strategy [60]. In the Borena pastoral region of Ethiopia, both retrospective outbreak records and seroprevalence data show that herd immunity levels are significantly below the 80% threshold recommended by FAO‐WOAH, with 53 outbreaks reported between 2018 and 2022 [21]. Although large‐scale seromonitoring conducted in the Indian state of Karnataka revealed that postvaccination seropositivity reached 73.4% in the 6–12 month age group and that ≥70% immunity threshold was achieved postvaccination in 69% of epidemiological units, the authors emphasize that >95% coverage campaigns and ≥80% sustained herd immunity are necessary for the national eradication target [57]. Modeling studies highlight not only nominal average coverage but also which subpopulations are vaccinated. Analysis considering the heterogeneous mixing in the Ethiopian lowland system has shown that the GCES’s proposed 70% coverage mass vaccination strategy may be insufficient for elimination if high‐risk pastoral and highly mobile herds are not adequately targeted, and that even with very high (>90%) coverage in low‐risk, more accessible sedentary herds, the virus may persist in high‐risk subgroups [55]. Unfortunately, this data shows that PPR vaccination campaigns have neither achieved the desired coverage in field conditions nor the desired level of herd immunity. Thus, unlike the success of high‐coverage, intensive campaigns in the rapid eradication of rinderpest, the scattered, resource‐depleted, and generally nonrisk‐based campaigns observed in many countries for PPR will not be sufficient for global eradication [12, 16, 55].

### 4.2. Surveillance, Diagnostics, and Underdetection

#### 4.2.1. Diagnostic Capacity and Current Laboratory Tools

At the laboratory level, a wide range of highly sensitive and specific diagnostic tools are available for PPRV diagnosis. Competitive ELISAs (c‐ELISA), which have been widely used in serological diagnosis, provide high sensitivity and specificity in field sera. The newer H protein‐based blocking ELISA (HPPR‐b‐ELISA) showed very high agreement with the c‐ELISA in field sera from multiple species [61]. In the same study, using the virus neutralization test (VNT) as the reference, HPPR‐b‐ELISA showed a sensitivity of 80% and a specificity of 96.36% [61]. The indirect ELISA (iELISA) developed in Pakistan, based on the local lineage IV isolate, presents a viable alternative for large‐scale serosurveys in developing countries, particularly due to its cost and storage advantages, exhibiting 90.6% sensitivity and 85.2% specificity when compared to c‐ELISA and 100% specificity and 82.1% sensitivity when compared to VNT [62].

A similar diversity is also evident in molecular diagnostic approaches. SYBR Green‐based RT‐qPCR targeting the conserved region in the F gene of PPRV achieved ~3 log lower detection limit compared to conventional RT‐PCR, detecting down to 13.6 copies, and also moderately increased the positivity rate in clinical sheep and goat samples [63]. TaqMan RT‐qPCR targeting the phosphoprotein (P) gene can detect all four genetic lineages (I–IV) of PPRV. The analytical sensitivity is 4 copies/µL for lineages II–IV and 40 copies/µL for lineage I. Compared to the previous N‐gene‐based RT‐qPCR method, it produces lower Ct values, especially in weakly positive samples, enhancing detection at low viral loads [64]. Among isothermal amplification approaches, two RT‐RPA‐based formats appear to be candidates for field use due to their ability to be cheaper and to provide results in a short time. In a recent study, real‐time RT‐RPA was able to generate a positive signal in ~3 min at 10^6^ copies per reaction and in ~7 min at 10^2^ copies per reaction at 40°C, with a detection limit calculated as 10^2^ copies per reaction with 95% probability. In the same study, the lateral flow strip format worked across a wide temperature range of 3745°C, with a faint test line visible from the 10th minute onwards, and the total test time remained under 25 min. The analytical sensitivity for LFS RT‐RPA was reported at the level of 150 copies, and no cross‐reactivity was detected with sheep and goat pox viruses, orf virus, and foot and mouth disease virus. These characteristics suggest that RT‐RPA could be used for rapid preliminary diagnosis in the field with portable devices or simple strip reading [65].

#### 4.2.2. Field Surveillance Gaps and Insufficient Recognition of Circulation

Despite these powerful tools, the utilization of diagnostic capacity in the field is limited by implementation gaps in many endemic countries. Regional road map meeting reports and assessments based on the PPR Monitoring and Assessment Tool (PMAT) framework developed by FAO and WOAH indicate that countries cannot timely report their data, and the reports frequently lack basic critical information such as sampling date and sample size, which weakens data accessibility and reliability [16]. Supporting regional assessments reveal that outbreak notifications, vaccination coverage, and surveillance outputs are not reported regularly and in an up‐to‐date manner in many countries, thus structurally hindering the timely and standardized transfer of laboratory data to decision‐making processes [12]. In many endemic environments, hard‐to‐reach pastoral areas, and regions with security concerns, sampling and reporting processes are delayed due to logistical obstacles, resulting in some suspected PPR cases remaining unconfirmed by laboratory testing and being inadequately captured by surveillance systems [12, 21]. For instance, recent field surveys mapping animal mobility and PPR risk in northern Nigeria had to severely restrict data collection to only a few accessible markets due to regional insecurity, directly demonstrating how conflict limits the data essential for accurate risk mapping and surveillance [66]. Serological field surveys provide a complementary lens on underdetection in such settings. In Punjab, Pakistan, a survey restricted to unvaccinated animals aged ≥6 months reported widespread seropositivity together with strong spatial heterogeneity, which is consistent with substantial circulation that may not be fully captured by routine case‐based surveillance [27]. Field evidence from North Shewa indicates that outbreak‐based risk classification alone may overlook areas with ongoing silent circulation or subclinical infection, supporting the integration of serological evidence into routine risk profiling [58, 59].

#### 4.2.3. Surveillance Challenges in the Later Stages of Eradication

The integration of diagnostic tools into eradication programs using the DIVA approach remains a programmatic gap. DIVA‐compatible differential ELISAs aligned with the Nigeria 75/1‐DIVA and Sungri/96‐DIVA vaccines have shown that infected and vaccinated animals can be distinguished serologically under experimental conditions [53]. However, large‐scale validation, standardization, and quality assurance are still required before these assays can be adopted for routine field use [53]. PMAT evaluations also point to the continuous need to strengthen surveillance and monitoring capacities across countries [12]. Taken together, these findings suggest that the diagnostic infrastructure required for highly sensitive tracking of infection chains and the detection of residual transmission foci in the final phases of eradication is not yet sufficiently developed.

Global and regional assessments indicate that there are significant geographical and institutional silent areas in PPR surveillance [12, 16]. According to analysis results based on FAO/WOAH databases and PMAT applications, covering 42 countries in Africa and Western Eurasia, 31% of countries in these regions still remain in Stage 1 (assessment) of the GCES, while 59.5% have reached Stages 2 (control) and 3 (eradication) [12]. Additionally, the absence of PPR cases in some countries indicates weak surveillance and reporting capacities rather than the actual disease‐free status [12].

Seroprevalence and retrospective outbreak analysis conducted in Ethiopia showed that despite 53 PPR outbreaks being reported in passive surveillance records during the 2018–2022 period, PPRV antibody seroprevalence was detected at 32.1% in unvaccinated herds and 45.5% in herds with unknown vaccination status, suggesting that passive surveillance may not fully reflect field circulation [21]. Data obtained from Nigeria also indicate that there may be a discrepancy between the reported data and the actual magnitude of circulation in the field [67]. Similarly, field data from the Nile Delta region of Egypt showed an overall PPRV seroprevalence of 30.5%, with substantially higher odds of seropositivity in unvaccinated animals and those sharing water sources, further illustrating how immunity gaps and management‐related risk factors can sustain field circulation [24]. The fragility of PPR‐free status in the absence of strict surveillance was strikingly demonstrated by the outbreak in Greece in July 2024; the introduction of lineage IV into a previously free European region highlights the constant threat posed by cross‐border movements [68]. The urgency of the threat has been further increased by the simultaneous outbreaks existed in Romania in July 2024; confirmed that the virus has breached the biosecurity barriers of multiple European Union member states [69]. The vulnerability of European surveillance and movement‐control systems was further illustrated by the continued expansion of PPR in Europe during 2025–2026. Following the 2024 outbreaks in Greece, Romania, Georgia, and Bulgaria, Hungary reported PPR in January 2025, while the first confirmed outbreaks were reported in Albania in June 2025 and in Kosovo in July 2025 near the Albanian border. Croatia subsequently reported its first PPR outbreak in December 2025. Updated outbreak assessments later documented additional outbreaks in Croatia and Albania during early 2026, indicating that PPR may still be circulating in affected areas despite the implemented control measures [70, 71]. This serves as a stark illustration of how formal biosecurity measures can fail in practice.

### 4.3. Genomic Epidemiology and Lineage Dynamics: Added Value Versus Scalability

Genomic epidemiology offers substantial solutions far beyond classical sero‐surveillance in deciphering the spatial and temporal dynamics of PPRV and makes significant contributions to refining eradication strategies [15, 72]. Current epidemiological data show that PPRV lineage IV has become dominant in both Africa and Asia [72–74]. The spatiotemporal shift of PPRV is visually striking when comparing the historical and contemporary distributions (Figure 1). Prior to 2015, lineages I, II, and III were historically confined largely to specific regions within sub‐Saharan Africa (Figure 1A). However, the trans‐continental dominance of lineage IV over the last decade is visually evident; this lineage now spans continuously from China and South Asia to the Mediterranean basin, expanding deeper into the African continent and recently breaching European borders (Figure 1B). Genomic data from 2025 confirm that isolates from India are clustered within lineage IV, showing 90%–100% nucleotide identity with other Asian strains [26]. This demonstrates the continued dominance of lineage IV in the Indian subcontinent and its rapid regional circulation [26]. Whole genome and partial gene sequencing have revealed that despite the historical predominance of lineage II in West Africa and lineage I in some pockets, lineage IV has rapidly spread over the last decade, occasionally replacing older lineages, and lineage IV has begun to suppress lineage II in West Africa [73–75]. In East Africa, molecular epidemiological data indicate heterogeneous lineage dynamics: Ethiopian samples collected after 2010 clustered within lineage IV, whereas complete genomes from Tanzania showed high nucleotide identity with lineage III viruses circulating in East Africa [76, 77]. A broad genomic dataset spanning from 2007 to 2024 in China shows that lineage IV entered the country through at least two independent introductions and evolved into a structure exhibiting temporal‐spatial correlation, diverging into seven clades over time [78].

Figure 1Spatiotemporal shift in the global distribution of PPR virus lineages (I–IV). Two distribution maps are generated to compare the pre‐2015 and post‐2015 periods using epidemiological and genomic data derived from the WOAH‐WAHIS database, PubMed, GenBank Nucleotide, and published literature. Countries highlighted in yellow represent regions with officially reported PPR cases but lacking publicly available genomic sequence data in GenBank for lineage assignment. (A) *Morbillivirus caprinae* pre‐2015 lineage distribution: historical boundaries where lineages I (dark blue), II (blue), and III (green) were predominantly localized within sub‐Saharan Africa. (B) *Morbillivirus caprinae* last 10 years PPR lineage distribution: lineage IV (red) exhibits a massive transcontinental expansion, effectively replacing historical lineages in multiple regions across North Africa and the Middle East, while recently breaching biosecurity barriers in Southeastern Europe.

**Figure:**
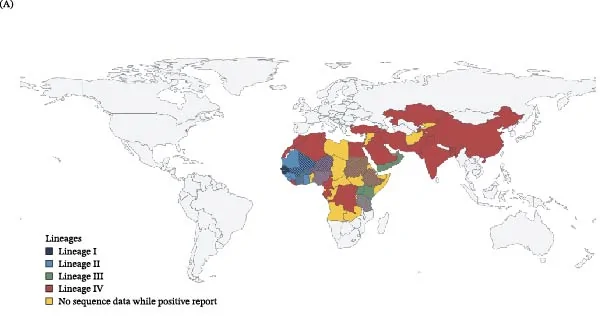

**Figure:**
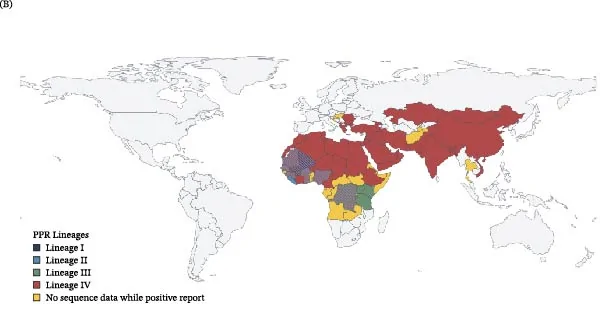

Phylogenetic and phylogeographic analyses are particularly valuable in mapping cross‐border spread pathways and reintroduction events. For example, genomic data from China show that lineage IV PPRV has been detected in both domestic small ruminants and several wild small ruminant species, indicating that the livestock–wildlife interface should be considered in genomic surveillance [78]. In examples at the Mali‐Senegal border in West Africa, cladistic clusters consistent with intense animal movements between neighboring countries and closely related sequences within the same lineage suggest that free trade and pastoral migration support a “source‐sink” type metapopulation structure [74, 75].

**Figure 2:**
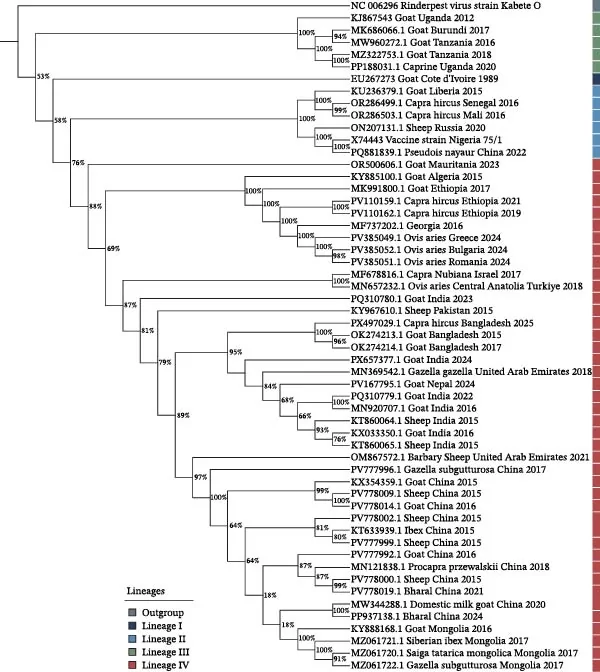
Phylogenetic analysis of *Morbillivirus caprinae* (PPRV) isolates complete genomes. Following multiple sequence alignment via MAFFT v7.526 [79], a maximum likelihood (ML) phylogeny was reconstructed utilizing IQ‐TREE. Node reliability was evaluated through 1000 ultrafast bootstrap iterations, and the final tree topology was annotated and visualized with iTOL v7.2.2 [80]. The tree demonstrates the genetic diversity and global dominance of lineage IV (red). Recent 2024 isolates from Southeastern Europe (Greece, Bulgaria, Romania) cluster tightly within this lineage, confirming a common origin for the recent European incursions. The tree is rooted using the rinderpest virus (Kabete O strain) as an outgroup.

In recent years, the introduction of portable sequencing technologies has made it possible to produce whole‐genome data under field‐oriented conditions even in low and middle‐income countries with limited infrastructure; in Tanzania, Oxford nanopore MinION sequencing enabled complete PPRV genomes to be generated from clinical samples within a few hours, supporting the mapping of lineage III circulation and pockets of endemicity [77]. However, sequencing costs, lack of bioinformatics expertise, and the need to clean erroneous/missing metadata in public databases cause genomic surveillance to remain project‐based, irregular, and narrow in scope in many endemic countries [72, 74]. For this reason, genomic epidemiology offers high added value, particularly in areas such as distinguishing reintroductions, evaluating suspicions of vaccine escape, resolving the connection between wildlife outbreaks and domestic populations, and defining regional eradication zones; however, to become a truly routine program tool, challenges of scalability, standardization, and sustainable financing need to be overcome [15, 74]. The evolutionary dynamics and host plasticity of PPRV are demonstrated in the phylogenetic analysis (Figure 2). The tree delineates the four historical lineages, illustrating the genetic divergence of lineages I, II, and III, which remain predominantly clustered within specific African sub‐regions. In contrast, lineage IV exhibits a higher degree of genetic diversity and a broad transcontinental expansion. The topology of the lineage IV clade indicates both its geographical distribution and its multihost capacity. Specifically, sequences from various wild ungulates intersperse with domestic small ruminant isolates from respective regions, supporting the role of the wildlife‐livestock interface in the epidemiology of the virus. Furthermore, within the lineage IV clade, the recent 2024 isolates from Southeastern Europe (Greece, Bulgaria, and Romania) form a distinct and tightly supported subclade, supporting a common origin for the European outbreak [70]. According to analyses presented by the EU reference laboratory for PPR, there was a 97%–99% probability that Romania was the first country infected in Europe, and the available sequences supported a common origin for the European outbreaks. This high similarity among available sequences highlights that the European PPR situation remains evolving and that the Black Sea basin continues to represent a critical interface for regional surveillance in Romania [70].

## 5. Structural Drivers Sustaining Transmission

### 5.1. Mobility and Trade Networks

It has been shown in numerous spatial‐epidemiological studies that PPR is closely associated with animal movements, especially in pastoral and semi‐pastoral systems. A classic field study examining the first introduction of PPR in southern Tanzania revealed that shared grazing, mixing of herds, and the sale of sick animals to other villages for slaughter at low prices were the main factors in the spread of the disease [81]. Studies conducted in Uganda have also shown that PPR seroprevalence is significantly associated with cross‐border (South Sudan and Kenya) animal trade, transhumance, and communal grazing [82]. A study conducted along the Tanzania‐Zambia border detected PPRV antibodies in small ruminants on both sides of the border and highlighted the need to further evaluate the effects of trade, proximity to borders, towns, and transport corridors on transboundary pathogen exposure [83]. This border‐related transmission model is consistent with recent findings from Uganda, where seroprevalence hotspots were identified, particularly along international borders with Kenya and Tanzania [23]. Additionally, uncontrolled interstate animal movements have been identified as the primary factor maintaining the high endemicity observed throughout Pakistan [28].

Network analysis based on the CILSS database in West Africa has revealed that cattle and small ruminant trade networks are extremely complex, mostly informal, and predominantly cross‐border in nature; approximately two‐thirds of live animals are subject to international trade [84]. In this network, it has been demonstrated that many hub markets are located near country borders, animal movements are concentrated along long‐distance corridors, and the majority of movements do not enter official records [84]. The high seroprevalence of FMD and PPR (~49% for PPR) found in cattle imported from Sudan to Egypt confirms the central role of live animal trade in the spread of transboundary diseases [85]. Beyond the epidemiological risks, the regulatory landscape further complicates control efforts. It has been shown that across East Africa, particularly at the Ethiopia‐Kenya border, cross‐border trade largely operates in an informal, legal‐illegal gray area where security forces and market actors play dual roles that both encourage and impede this trade; animal health certification and quarantine mechanisms largely fail to cover these flows [86]. Under these conditions, it is difficult to fully implement classical eradication approaches based on official quarantine and mobility restrictions in the field.

Seasonal transhumance in pastoral and agro‐pastoral systems leads to long‐distance movement of herds for water and pasture, and high contact rates between different herds at common grazing areas and water points; this contributes to higher seroprevalence and risk of transmission in pastoral herds compared to sedentary systems [55]. The intensive mixing of herds from different regions at market and grazing nodes creates a persistent susceptible host pool for PPRV, supporting endemicity [55, 87].

Modeling studies show that if high‐risk pastoral or nomadic herds and high‐contact nodes remain unvaccinated, PPRV circulation can continue even if the overall vaccination rate exceeds 90%, whereas risk‐based vaccination focused on high‐contact populations and nodes significantly increases the likelihood of eradication [55]. This is empirically supported by a recent spatial multicriteria decision analysis (MCDA) in northern Nigeria [66].

PPR, by its nature, is a transboundary disease affecting regions with dense sheep and goat populations in Africa and Asia; the repeated introduction through trade, pastoral mobility, and market networks may allow endemicity to persist despite domestic control efforts [87, 88]. GCES and current modeling studies therefore recommend placing movement patterns and network nodes at the core of control strategies, and implementing risk‐based planning at the scale of high‐contact populations and trade/movement nodes rather than at the herd level [55, 66].

### 5.2. Fragility, Conflict, and Programme Disruption

Historical and contemporary evidence indicate that armed conflicts can disrupt the transmission dynamics, surveillance, and control of infectious diseases. In conflict‐affected parts of the Eastern Mediterranean Region, the re‐emergence of vaccine‐preventable human diseases such as measles and polio has been linked to interruptions in vaccination activities and to forced population movements that created new epidemiological connections [89, 90].

Although conflict‐focused analyses specific to PPR remain limited, available evidence suggests that veterinary services have been substantially weakened in endemic settings such as Syria, Yemen, Iraq, and the Sahel belt. At the same time, the erosion of livestock‐based livelihoods appears to have contributed to increases in uncontrolled animal sale, movement, and slaughter [10, 12, 91, 92]. A review focused on the Middle East further noted that conflict in Syria and neighboring countries weakened, and in some areas rendered nonfunctional, surveillance networks, thereby limiting any reliable assessment of the field situation of PPR [17]. Large‐scale geopolitical crises may also affect PPR control indirectly by placing additional pressure on animal health systems. The Russia‐Ukraine war and the Israel‐Gaza conflict are examples of situations in which disruptions in food and feed supply chains, together with pressure on national budgets, may reduce the resources available for veterinary services and disease control [93]. In Gaza, livestock production has been severely affected, with FAO reporting that almost 95% of cattle had died, while only around 43% of sheep and 37% of goats remained alive by September 2024 [94]. A subsequent FAO response described severe shortages of essential veterinary materials and the need for veterinary kits, fodder, vaccines, and animal health inputs to protect remaining livestock and sustain herding livelihoods under constrained humanitarian access [95, 96]. Given the broader deterioration of surveillance capacity across parts of the Middle East [17], the sustainability of systematic eradication activities under such conditions appears highly uncertain. In this context, the framing of PPR eradication as an International Public Good highlights the need for continued resource mobilization even in fragile settings, particularly to reduce the risk of persistent virus reservoirs with wider regional and trade implications [5].

### 5.3. Social and Environmental Pressures

Small ruminants constitute an important productive asset for women in many rural areas of Africa and Asia, and PPR therefore has direct implications for women’s livelihoods and for household food and nutrition security [97, 98]. Studies examining PPR vaccine value chains through gender and intersectional frameworks in Nepal, Senegal, and Uganda have shown that women often face systemic barriers to accessing vaccines and veterinary services. These barriers may be further intensified by ethnicity, caste, and geographical remoteness. In Nepal, Uganda, and Senegal, context‐specific social structures such as caste hierarchy, ethnicity, and settlement remoteness intersect with gender in ways that shape access to PPR vaccination services and participation in campaigns [97–99].

Qualitative studies from Uganda and Senegal illustrate how these intersecting inequalities operate in practice. In these settings, the predominance of local male actors within vaccine supply chains may restrict women’s access to both vaccines and campaign‐related information [97, 99]. Taken together, these findings suggest that PPR vaccination programmes should not only monitor coverage among those reached but also consider which groups remain excluded and should develop targeted delivery approaches for herds managed by women [97–99].

Climate change may also indirectly influence PPR epidemiology through increasing pressure on the pastoral production systems. Projections across Africa suggest that, by 2050, the climatic boundary between agro‐pastoral and pastoral production systems may shift in favor of the pastoral systems, potentially reducing the current 4.43 million km^2^ agro‐pastoral belt by 27%–81% [100]. Such changes are associated with increased production vulnerability and livelihood stress, particularly in marginal areas exposed to drought and water scarcity [100]. In parallel, pasture degradation, feed insufficiency, and limited institutional support may increase livelihood stress and alter pastoral mobility patterns, with indirect implications for disease‐control planning in mobile herds [101]. Field‐based MCDA risk mapping in northern Nigeria provides further support for the relevance of these environmental and mobility‐related constraints by identifying climate, small ruminant density, proximity to dry areas, commercial movements, and transhumance movements as drivers of PPR occurrence [66]. Broader African evidence indicates that reduced rainfall in transhumant pastoralist territories can alter seasonal mobility and increase conflict risk in neighboring agricultural areas, with implications for both pasture‐related conflict and new contact opportunities relevant to transboundary animal diseases [102]. Although these studies are not specific to PPR, they describe ecological and behavioral shifts, under climate stress that may facilitate the spatial spread of infectious diseases more broadly [101, 102]. Relatedly, resource competition and associated local conflicts may constitute an additional indirect risk factor, both by altering seasonal movement patterns and increasing contact between the pastoral and agricultural systems [102].

Taken together, these observations suggest that climate‐related pressures may complicate PPR eradication in at least two ways: by increasing the dynamism and fragility of pastoral systems, and by exposing veterinary infrastructure to additional environmental stress. Under such conditions, technical interventions alone may be insufficient to achieve the 2030 global targets. These considerations suggest that sustained progress is likely to depend not only on veterinary measures but also on broader alignment with climate policy, land‐use management, and conflict mitigation mechanisms.

## 6. Synthesis and Pathways Beyond 2030

### 6.1. Feasibility Synthesis for the 2030 Eradication Target

Several of the biological and technical features that supported rinderpest eradication are also present in PPR, including a single serotype, the availability of reliable laboratory diagnostics, strong and long‐lasting immunity induced by live attenuated vaccines, and predominantly direct‐contact transmission within a relatively limited host range [3, 16, 50]. However, the feasibility of meeting the 2030 target appears to depend less on intrinsic virological constraints than on the performance of delivery systems. Recent studies from Ethiopia support this interpretation by showing that repeated vaccination efforts can still be undermined by persistent operational bottlenecks and incomplete flock‐level immunity, thereby limiting progress toward effective interruption of transmission [58, 59].

From an eradication perspective, the 2030 target can be viewed as a synchrony problem: residual transmission foci must be eliminated faster than they are reseeded through transboundary movement. The outbreaks reported in Greece and Romania in July 2024 illustrate how disease‐free areas may still be breached when surveillance and movement controls are insufficient, and infected animals, or contaminated trade pathways, reach susceptible populations [68, 69].

The evidence reviewed here suggests four related reasons why achieving the 2030 target globally remains difficult.

First, immunity cannot be assessed solely in terms of average coverage; sustained postvaccination immunity in key subpopulations is equally important. Although theoretical herd immunity thresholds for PPR are often framed around *R*_0_‐based targets, commonly around 70%–80% and higher in high‐risk settings, several modeling studies indicate that heterogeneity in the contact structure and vaccination access may allow circulation to persist even where national coverage appears adequate [54–56]. Pastoral and mobile production systems may function as metapopulation reservoirs, reintroducing infection into more accessible sedentary systems [54].

Second, the endgame is likely to be constrained by the surveillance performance. In the elimination phase, the limiting factor may shift from vaccine availability to the sensitivity, timeliness, and representativeness of surveillance systems. PMAT‐based assessments suggest that many countries still lack consistent, high‐quality surveillance outputs and complete metadata, which weakens risk‐based decision‐making and delays the identification of residual foci [12, 16]. In such settings, the risk of premature claims of freedom may increase while low‐level transmission remains undetected.

Third, operational fragility continues to limit effective delivery at scale. Although currently available vaccines are efficacious, thermolability and dependence on cold‐chain systems remain important operational constraints, particularly in the hottest and most remote endemic settings. Advances in thermotolerance criteria and stabilizer development show what may be technically achievable, but wide‐scale implementation is not yet routine in many national programmes [16, 51]. A comparable gap is also evident for DIVA‐based approaches [12, 53].

Fourth, transmission is shaped by structural drivers beyond the veterinary sector. Mobility and trade networks, conflict‐related disruption, and climate‐related changes in pastoral movement patterns may all reduce campaign continuity and increase the risk of reintroduction [17, 86, 100, 102].

Taken together, these points support a cautious interpretation. PPR eradication remains technically feasible, but achieving it everywhere by 2030 appears unlikely unless delivery performance, surveillance sensitivity, and cross‐border coordination improve substantially and in parallel across endemic regions. In that sense, 2030 may be better viewed as a mobilizing milestone linked to measurable performance indicators rather than as a strict endpoint.

### 6.2. Alternative Pathways Beyond 2030: A Pragmatic Road Map

A pragmatic post‐2030 road map will likely require a shift from primarily local interventions toward stronger regional coordination. The Pan‐African Programme for the Eradication of PPR may provide a useful model for other endemic regions [18]. As reflected in the FAO GEP II and III plans, access to vaccine banks and adherence to WOAH Terrestrial Code standards for safe commodity trade, are likely to remain important components of this process [13, 103].

Accordingly, a post‐2030 road map may be framed around three linked objectives: reducing transmission in high‐connectivity reservoirs, increasing the probability of detecting residual foci, and limiting reintroduction through coordinated regional governance. In the first phase, covering ~2026–2030, the emphasis may need to remain on quality at scale and on reducing structural blind spots. This could involve moving from nominal mass vaccination toward risk‐based planning that explicitly targets pastoral reservoirs, border corridors, and major market hubs while using movement calendars such as transhumance and market peaks to time campaigns to periods of highest mixing risk [55, 82, 84]. Postvaccination seromonitoring may also need to be institutionalized as a decision tool rather than treated mainly as a reporting exercise, with PVIR thresholds defined by the risk stratum and rapid mop‐up campaigns initiated whenever immunity falls below target levels [56, 60]. Wildlife risk should likewise be integrated more systematically, with emphasis on high‐intensity livestock‐wildlife interfaces and large susceptible wild populations rather than universal wildlife surveillance [19, 41]. This phase may also provide a suitable window for broader field evaluation of newer vaccine technologies, including heat‐stable formulations and DIVA‐related approaches, together with associated diagnostic tools.

In a second phase, ~2030–2035, programme priorities may shift toward endgame targeting and regional verification. Broad campaigns could be progressively replaced by ring‐, buffer‐, and corridor‐based vaccination strategies around detected foci and high‐risk borders, supported by rapid response teams and prepositioned vaccine capacity. At the same time, surveillance systems would need to approach endgame‐level sensitivity through stronger routine molecular confirmation, improved metadata completeness, and wider deployment of field diagnostics where laboratory access remains limited [16, 64]. Where feasible, DIVA‐compatible approaches might be piloted in late‐stage settings to support serological verification of freedom, although wider scale‐up would still depend on adequate field validation, quality assurance, and cost–benefit justification [12, 53].

In a third phase, beyond 2035, the main priority is consolidation of freedom and prevention of re‐emergence. Under such conditions, the campaign‐based approach would need to give way to long‐term freedom‐maintenance systems combining border biosecurity, risk‐based surveillance, and rapid outbreak response capacity. Trade incentives may also need to be aligned more closely with compliance, including the implementation of safe commodity pathways and standards‐based trade rules designed to reduce illegal animal movement and related risks to disease freedom [103].

More broadly, eradication beyond 2030 may need to be treated as a regional public good supported by pooled financing, harmonized targets, and shared accountability. The Pan‐African Programme for the Eradication of PPR illustrates how political ownership and large‐scale financing can be mobilized; comparable regional arrangements may be necessary in other endemic areas to prevent the persistence of last‐mile reservoirs [5, 18]. Overall, the most realistic pathway beyond 2030 may not be a relaxation of ambition but a reframing of how that ambition is operationalized.

## 7. Conclusions

Although the global objective of PPR eradication remains scientifically sound, the 2030 deadline is constrained by the convergence of several delivery bottlenecks, including the difficulty of sustaining ≥80% immunity in high‐mobility reservoirs, closing surveillance blind spots, and reducing reseeding through cross‐border movement. The available evidence suggests that programme delay or failure is less a consequence of insufficient vaccine efficacy than of the difficulty of delivering and verifying elimination in fragmented, conflict‐affected, and informally connected settings [12, 17, 84].

Accordingly, the most defensible interpretation of the 2030 target is as a milestone that accelerates national progression under GCES and PMAT, rather than as a universal endpoint for verified global freedom. Countries and regions that achieve freedom will still require robust maintenance systems because the reinvasion risk remains persistent, as demonstrated by recent European outbreaks [70, 71].

Therefore, a good practical approach covers prioritizing risk‐based vaccination in high‐connectivity regions, surveillance systems that detect low level of virus circulation, enhanced cross‐border coordination, and integrated equity and climate resilience into delivery models. Under these conditions, a post‐2030, staged pathway can achieve durable global freedom from PPR.

## Author Contributions

The scientific content, interpretation of the literature, selection and verification of references, and conclusions were performed by the authors.

## Funding

This study is performed under the spect of the research project funded by the Bursa Uludag University Research Fund (Grant TGA4‐2025‐2359).

## Disclosure

The final approval of the manuscript was performed by the authors.

## Ethics Statement

This study does not involve human or animal subjects; therefore, ethical approval was not required.

## Conflicts of Interest

The authors declare no conflicts of interest.

## Data Availability

Data sharing is not applicable to this article as no datasets were generated or analyzed during the current study.
